# Formulating Diets for Improved Health Status of Pigs: Current Knowledge and Perspectives

**DOI:** 10.3390/ani12202877

**Published:** 2022-10-21

**Authors:** Lucas A. Rodrigues, Bonjin Koo, Martin Nyachoti, Daniel A. Columbus

**Affiliations:** 1 Prairie Swine Centre, Inc., Saskatoon, SK S7H 5N9, Canada; 2 Department of Animal and Poultry Science, University of Saskatchewan, Saskatoon, SK S7N 5A8, Canada; 3 Department of Animal Science, University of Manitoba, Winnipeg, MB R3T 2N2, Canada

**Keywords:** health, immunity, metabolism, nutrition, swine

## Abstract

**Simple Summary:**

Today’s swine production systems are mostly intensive, and pigs are continuously exposed to pathogens and immune stimulatory antigens that may negatively impact productivity. It is known that immune system stimulation may reduce appetite, growth, and nutrient use efficiency compared to healthy animals. On the other hand, there is progressive pressure towards the reduction in antibiotic usage in livestock production, which highlights the need for furthering our understanding of the relationship between nutrition and the immune response. Swine nutritionists will need to consider the role of nutrition on health in order to develop programs that support production and robustness of pigs under a variety of stressful conditions. Among the nutritional strategies whose utilization may directly impact on the immune status of pigs, feeding low protein, amino acid-supplemented diets, supplementation of functional amino acids, dietary fiber level and source, diet complexity, organic acids, and plant secondary metabolites are at the forefront. As such, this review will discuss the impact of immune status on swine production and the interaction between nutrients and animal health, focusing on the roles of each nutritional strategy during times of immune challenge.

**Abstract:**

Our understanding of nutrition has been evolving to support both performance and immune status of pigs, particularly in disease-challenged animals which experience repartitioning of nutrients from growth towards the immune response. In this sense, it is critical to understand how stress may impact nutrient metabolism and the effects of nutritional interventions able to modulate organ (e.g., gastrointestinal tract) functionality and health. This will be pivotal in the development of effective diet formulation strategies in the context of improved animal performance and health. Therefore, this review will address qualitative and quantitative effects of immune system stimulation on voluntary feed intake and growth performance measurements in pigs. Due to the known repartitioning of nutrients, the effects of stimulating the immune system on nutrient requirements, stratified according to different challenge models, will be explored. Finally, different nutritional strategies (i.e., low protein, amino acid-supplemented diets; functional amino acid supplementation; dietary fiber level and source; diet complexity; organic acids; plant secondary metabolites) will be presented and discussed in the context of their possible role in enhancing the immune response and animal performance.

## 1. Nutrition and Health: The Merge

Current nutrient requirement estimates are largely based on growth performance response, however, nutrient effects on non-proteinogenic functions (e.g., intestinal development, immune status, and response) has been increasingly demonstrated. With legislation introduced in some countries to eliminate use of antimicrobial growth promoters and increasing pressure to reduce overall antibiotic use in livestock production, it will become increasingly important to understand how immune status affects nutrient requirements and how feed formulations can be adjusted to support animal robustness in addition to growth performance. The impact of disease-challenge can be substantial. For example, pigs kept in low sanitary conditions (LSC; e.g., lack of vaccination, environmental hygiene and biosecurity protocol, and preventive antibiotic injection) having a 55 g/d reduction in daily gain during the complete fattening period compared to counterparts housed in high sanitary conditions (HSC; [1]). This indicates a high cost associated with the activation of the immune system, including a higher production of immune cells and signaling molecules as well as losses in efficiency of affected tissues, which will decrease the efficiency of nutrient utilization for body protein deposition [2]. It is reasonable to infer that nutritionists must consider the components associated with building a strong immune system when formulating swine diets in commercial operations. Of note, while nutritional strategies may support animal robustness during a disease and recovery from illness, attention should also be given to dietary strategies that trigger excessive immune activation and may impair the efficiency of animal production.

Additional evidence for the importance of nutrition for immune status lies in the endocrine regulation of appetite and growth. Specifically, the reduction in performance due to immune system activation is not entirely, and not always, associated with a reduction in feed intake. Upon exposure to stressful situations, mononuclear myeloid cells of the innate immune system synthesize and release proinflammatory cytokines such as interleukin (IL)-1b, IL-6 and tumor necrosis factor-α (TNF-α) [3]. These cytokines will orchestrate (1) a redirection of nutrients from growth towards the immune system [3,4], (2) a specific response of the central nervous system with fever, decreased feed intake, and activation of hypothalamic–pituitary–adrenal axis [4,5], (3) a suppression of gastric emptying and motility [4], and (4) an increased concentration of glucagon, insulin, and leptin in blood [6]. These events combine to affect feed intake and weight gain, and guide how inflammation impacts growth.

In order to meet growing demand, pork production will need to increase its sustainability and efficiency, which will encompass the development of nutritional programs which support animal growth performance and robustness under commercial conditions. Several nutritional strategies have been identified as potential governors of the interaction between growth and health in pigs, including feeding low protein (LP) diets, functional amino acid (FAA) supplementation, and provision of fiber sources and natural (e.g., phytochemicals) compounds. As such, the present review will discuss the impact of different immune stimulating conditions on nutrient digestion and metabolism and how different nutritional interventions may help nutritionists formulate diets for improved health status of pigs. One important aspect approached by the present review is the differentiation between (1) situations where stressors decrease feed intake and consequently impair growth performance vs. (2) the effects of immune system stimulation on nutrient metabolism.

## 2. Immune System Stimulation, Nutrient Efficiency and Requirements

Immune stimulation and inflammation result in a number of effects in the pig, most notably a reduction in voluntary feed intake and efficiency of nutrient utilization, even in the absence of clinical manifestation of disease [7], although this may not always be the case. While both feed intake and nutrient utilization efficiency influence pig performance, the extent to which reductions in these factors are related to the reduction in performance is dependent on the specific immune challenge [8]. For example, Rodrigues et al. [9] showed that the reduction in growth in challenged compared with control pigs associated with a reduction in feed intake was 14, 4, 45, 1, and 1% in pigs challenged with enteric pathogens, environmental stressors, bacterial lipopolysaccharide (LPS), respiratory pathogens, and sanitary conditions, respectively. This is important to note as, in experimental environments, several challenge models are used to investigate stimulation of the immune system and the associated effects on outcomes such as growth performance impairment, nutrient requirements, and immune status. These include challenge with enteric [10,11] and respiratory pathogens [12,13], housing pigs under heat stress, high stocking densities, or suboptimal sanitary conditions [1,14,15,16,17], and triggering systemic immune stimulation using LPS [18]. Overall, it is likely that the effect of any nutrition intervention in challenged pigs will be, at least in part, due to the specific challenge model used and results may not be transferable to other conditions.

Another important factor differentially impacting performance response in challenged pigs is the stage of production. For example, the meta-analytical approach of Rodrigues et al. [9] demonstrated that recovery of performance in post-weaned pigs was dramatically influenced by the recovery of feed intake, which was not observed in nursery and grower phases. The authors also reported a more abrupt and greater decrease in both growth and feed intake in younger, lighter pigs compared to older, heavier pigs. This may be explained by (1) the impact of gut fill, where newly weaned pigs take longer to reach maximum feed intake and (2) decreased growth potential due to limited feed intake in the immediate post-weaning phase [19]. The nutritionist needs to understand the many stressors concomitantly reducing post-weaning feed intake, including introduction to solid feed [20], abrupt depletion of passive immunity transfer from the dam [21,22], and the building of a new hierarchy with unfamiliar littermates [23]. Under this scenario, reestablishment of nutrient intake through higher nutrient supply or recovery of feed intake will be one of the most important governors for restoring weight gain [24].

Since different stressors may impact productivity at different stages of production, it is pivotal to understand how the exposure to pathogens and other stressors changes nutrient utilization and the associated decrease in performance. According to Pluske et al. [25], it is possible to manipulate the immune system through nutrition and other supportive strategies, including suppressing the presence and action of pathogens, breeding for improved resilience, and controlling the immune system to prevent overt response. With the increased trend for reduction in in-feed antimicrobial growth promoters’ usage, and the need for a more sustainable production system, a multidisciplinary approach will be needed to attenuate the negative impact of stressful agents, which are expected to be more harmful as pork production becomes more efficient [26]. The next sections will address the specific effects of individual challenge models on immune system activation and the associated effects on nutrient digestion and metabolism and build a relationship with growth performance measures. Subsequently, key nutritional strategies with proven benefits on the ability of pigs to cope with challenge models, including LP, AA-supplemented diets, FAA supplementation, dietary fiber level and source, diet complexity with inclusion of highly digestible ingredients, organic acids (OA), essential oils (EO), and natural compounds will be discussed.

**Figure 1:**
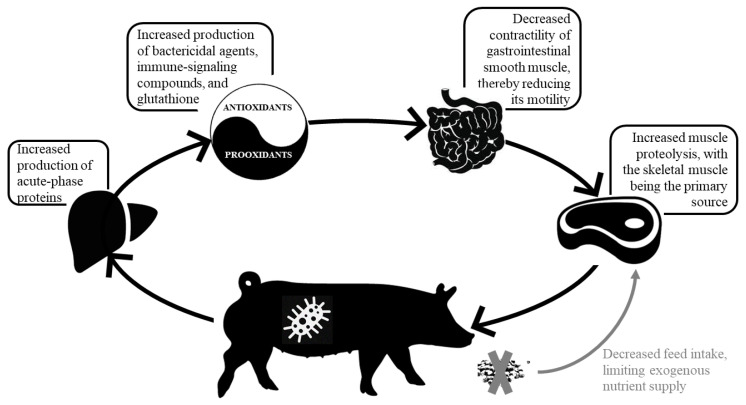
Pigs subjected to stimulation of immune system experience increased production of acute-phase proteins (e.g., C-reactive protein, serum amyloid A, haptoglobin, and pig-MAP). Additionally, there is a marked syntheses of endogenous antioxidants to mitigate oxidative stress, including the production of immune-signaling compounds, the release of superoxide dismutase by phagocytic cells and the increased turnover of glutathione, which is a key, non-enzyme antioxidant in the body. Furthermore, a higher concentration of circulating pro-inflammatory cytokines decreases the contractility of gastrointestinal smooth muscle, thereby reducing its motility and sparing energy and nutrient expenditure for digestion. In attempt to minimize metabolic costs, pigs mobilize nutrients, particularly amino acids, from body reserves. This is mediated by proinflammatory cytokines and reactive oxygen species, which stimulate the IκB/NF-κB signaling pathway, eventually activating the ubiquitin proteasome system, and further triggering muscle proteolysis, with the skeletal muscle being the primary source. The reduced feed intake, which is mainly a result of upregulation of feed intake-lowering (anorexigenic) peptides and downregulation of orexigenic peptides, limits the exogenous nutrient supply and aggravates muscle proteolysis.

Stimulation of the immune system results in higher concentration of circulating acute-phase proteins (e.g., C-reactive protein, serum amyloid A, haptoglobin, pig-MAP) [27], immune cell proliferation (e.g., clonal lymphocyte and monocyte differentiation), secretion of various molecules secreted by immune cells (e.g., cytokines, immunoglobulins), and lymphoid tissue hyperplasia [7] (Figure 1), with AA necessary to support the activated immune system. Although the amount of AA required for the activated immune system is not a substantial component of whole-body protein turnover, the AA profile required to support the immune response is drastically different than for muscle protein synthesis [28,29], resulting in a disparity between AA requirement and supply and reduction in utilization efficiency. Another significant expenditure of AA during inflammation is associated with the syntheses of endogenous antioxidants to cope with oxidative stress. Although the mitochondrial electron transport chain is a major site of free radical production, immune cells, such as phagocytes and monocytes, produce free radicals in the form of bactericidal agents (e.g., O_2_ and HOCl) or during the process of producing immune-signaling compounds (e.g., leukotriene and nitric oxide) [30]. For example, phagocytic cells, in a process called respiratory burst [30], consume large amounts of oxygen during phagocytosis and rapidly release superoxide (via the activation of NADPH oxidase) into the phagosome to kill bacteria. Likewise, a tripeptide γ-glutamyl-cysteinyl-glycine or glutathione (GSH) is a key, non-enzyme antioxidant in the body, and its turnover increases in response to ISS. Indeed, studies using isotope tracer have confirmed that the LPS-induced ISS accelerated transsulfuration pathway, converting Met to Cys and increasing Cys metabolism, mainly due to synthesizes of GSH in pigs [31].

Therefore, it is reasonable to infer that traditional AA requirements are not applicable to immune-activated pigs because they were determined for optimal growth (protein accretion) under normal physiologic conditions [32]. Accumulating evidence has shown that dietary supplementation of specific AA can strengthen the pig’s immune system by minimizing body protein loss and accelerating its recovery [32]. This strategy seems to be more relevant for nursery pigs, who commonly undergo ISS post-weaning period.

## 3. Immune Challenges in Pork Production

### 3.1. Enteric Pathogen Challenge

The most common enteric diseases at pig farms are salmonellosis and swine enteric colibacillosis characterized by neonatal diarrhea and post-weaning diarrhea. The respective causal pathogens of these diseases are *Escherichia coli* and *Salmonella* spp. Of note, pathotypes F4+ and F18+ of enterotoxigenic *Escherichia coli* (ETEC) are the most predominant pathogens involved in post-weaning diarrhea [33]. Although colibacillosis is a major concern in nursery production, salmonellosis caused by infection of *Salmonella* enterica, particularly the serovar Typhimurium (ST), is widely observed throughout pork production [34,35]. In-feed antimicrobial growth promoters have been widely used to prevent or suppress the effects of enteric diseases, however, due to the ban on their use in many jurisdictions, animal nutritionists have made great efforts to seek alternative means to enhance gastrointestinal health while reducing antibiotic use. Although enteric pathogens have different pathotypes and pathogenicity, they all infect intestinal epithelial cells, triggering the action of the host’s immune system, generally leading to diarrhea [33]. For example, the ETEC flagella attach to the carbohydrate moieties of non-acid glycosphingolipids and glycoproteins present on the small intestinal epithelial cell layer, where they produce toxins (heat-labile and heat-stable toxins) (Table 1). These toxins activate the cyclic guanosine monophosphate and cyclic adenosine monophosphate systems, resulting in disruption of chloride and ion channels and, eventually, osmotic diarrhea [36]. Similarly, ST attaches to the intestinal epithelial cells and delivers a specialized set of effectors. Subsequently, ST injects *Salmonella* invasion protein into the intestinal epithelial cells, leading to cytoskeletal rearrangement in host cells, disruption of the normal epithelial brush border, and activation of the immune system [37]. The infected cells lose electrolyte absorption capabilities, thereby leading to diarrhea [38].

Intestinal inflammation and disrupted integrity are common observations in pigs challenged with ETEC or ST. Inflammation and redox signaling systemically govern the fate and permeability of intestinal epithelial cells, which are relevant to intestinal integrity [39,40]. Pro-inflammatory cytokines trigger mitogen-activated protein kinase signaling and lead to apoptosis of enterocytes on the villus, mainly at the tip [41]. Furthermore, the expression of myosin light-chain kinase, which causes cytoskeletal contraction, is activated under inflammatory conditions, thereby decreasing intestinal permeability [42]. Pigs challenged with ETEC or ST are characterized by poor intestinal morphology (e.g., villus height, villus width, and crypt depth) and permeability, as well as reduced brush border enzyme activity and nutrient transporter expression compared to non-challenged counterparts [43,44,45]. Indeed, ETEC-challenged pigs have lower ileal digestibility of nutrients (e.g., AA) than sham-challenged control pigs [46].

As enteric pathogens eventually stimulate the systemic immune system, the metabolic pathways change. These changes are initiated by the immediate increase in concentration of acute-phase proteins (e.g., haptoglobin) and immunoglobulins following pathogen exposure [10,44]. As a result, the requirements for several AA such as Thr, Trp, and sulfur AA (SAA), may be elevated for optimal growth performance and immune response [47,48,49].

### 3.2. Respiratory Pathogen Challenge

As a result of intensive, confined systems of modern pig production, respiratory diseases continue to be a cause of concern in pork production, commonly resulting in impaired weight gain and feed efficiency, poor animal welfare, and increase in medication costs [74]. There are several reports from different parts of the world indicating significant prevalence of pneumonia (20 to 80%) and pleuritis (4 to 60%) in slaughtered pigs, which indicates an important impairment of the respiratory tract throughout the productive life [51,52,53] and potentially significant productive costs of immune stimulation. The term porcine respiratory disease complex (PRDC) is often used to address the several bacteria, viruses and mycoplasmas involved in the etiology of respiratory diseases [75,76]. The most important agents are *Actinobacillus pleuropneumoniae*, *Mycoplasma hyopneumoniae*, *Pasteurella multocida*, porcine circovirus type 2, porcine reproductive and respiratory syndrome virus (PRRSV), porcine respiratory coronavirus, and *Salmonella* choleraesuis. The growth performance response of pigs to respiratory pathogens will be dependent on the agent involved. Two recent meta-analyses showed similar results related to severity of respiratory challenge [8,9]. In general, respiratory diseases were less detrimental to performance than the other immune challenges, which may possibly be due to a greater degree of compensatory growth following respiratory infections [77]. Moreover, many respiratory pathogens produce an immune response that is more likely to be contained within the affected tissues (e.g., neutrophilia and damage to cilia [54]), resulting in more minor clinical response and a reduction in a significant response in growth performance. However, subclinical infections can still result in significant economic consequences. For example, an economic analysis indicated that an increase in lung lesions above 15.1% resulted in a loss of up to $6.55 per pig at slaughter.

Among respiratory pathogens, there is more evident information in the literature to show that PRRSV is the most detrimental to performance. For example, body weight of weaned barrows was decreased by 5% in PRRSV-infected compared to non-infected pigs [78]. This is in agreement with Schweer et al. [12] who reported a 10% reduction in weight gain, 6% reduction in feed intake, and 7% reduction in feed efficiency in PRRSV-infected pigs compared to non-infected pigs through a market weight of approximately 128 kg. In order to assess whether the reduction in performance is driven by decreased feed intake and/or nutrient metabolism, Helm et al. [57] compared PRRSV naïve, ad libitum fed, PRRSV-inoculated, ad libitum fed, and PRRSV naïve, pair-fed to the PRRSV-inoculated pigs’ daily feed intake. Growth performance and feed efficiency were negatively impacted by PRRSV infection compared to the pair-fed group which indicates modification in nutrient metabolism. Specifically, markers of skeletal muscle synthesis (e.g., phosphorylation of protein synthesis markers) were reduced while liver glycogen was more depleted in PRRSV-infected compared to the pair-fed group. This also agrees with a 13% reduction in both whole-body protein accretion when pigs are infected with PRRSV, which reduced carcass lean yield [12]. A recent study by Schweer et al. [79] further demonstrated that PRRSV infection dramatically reduces weight gain (30%) and feed intake (26%) which was not accompanied by a decrease in the apparent total tract digestibility (ATTD) of AA and energy. This provides evidence that PRRSV infection reduces growth performance through a combination of reduced feed intake and decreased nutrient utilization.

### 3.3. Degradation of Sanitary Condition

While it is important to understand the effects of individual pathogens on pig immune status and performance, in commercial settings, pigs are more likely to be exposed to a variety of pathogens. This exposure is the result of an increase in environmental pathogen load. Moreover, the response of pigs to pathogens is complicated in commercial settings through the use of antimicrobial growth promoters and vaccination protocols which reduce pathogen load and enhance herd immunity to pathogens and individual pig ability to avoid infection. Experimentally, sanitary challenge represents an increase in pathogen exposure via absence of cleaning and disinfection protocols, reduction in vaccination, reduction in antibiotic use, or a combination [2,15,80]. Pigs are exposed to various stressors, broadly classified as non-biological stressors (e.g., noxious gases, dust) and biological stressors (e.g., bacteria, virus). The sanitation challenge aims to mimic the practical commercial swine barn environment and mildly activate the pig’s immune system and is thus often regarded as subclinical compared to studies of other pathogen, toxin, and stress challenges. Nevertheless, this model has been advantageously adopted by studies that tested diet interventions for pigs raised in commercial environments [63] and in which nutrient requirements of pigs housed in experimental or commercial environments were compared [15]. Because the sanitation challenge model can be applied without special facility or inoculum preparation, the experimental design (e.g., treatments, replications) is more flexible than that of other challenge models. Due to this design flexibility, the model can be applied to a large number of pigs, which increases the statistical reliability of growth performance (weight gain and feed intake).

Because LSC are usually created by the accumulation of manure in the pens, the pigs are more likely to ingest pathogens from the manure orally. Thus, gut health indicators, such as intestinal integrity and microbial composition, are typically investigated in sanitary challenge studies [61,62]. Furthermore, pathogens or dust can be transferred into the body through the nasal tract, so respiratory health indicators are also of interest in these studies [1]. Indeed, LSC increased the incidence of respiratory tract inflammation, possibly because of the increased loads of respiratory pathogens [1]. Additionally, LSC are often characterized by the buildup of noxious gases, such as H_2_S and NH_3_, as well as dust [80,81], which possibly irritate the respiratory tract. Pigs raised in LSC generally have lower body weight gain compared to those raised in high sanitary conditions [1,60,64,81]. However, whether this low body weight gain is accompanied with low feed intake has been argued. Moreover, it has been consistently reported that intestinal inflammation, oxidative stress, and disturbed intestinal integrity are consequences of housing pigs in LSC. For example, LSC also upregulates the expression of inflammatory cytokines, such as interferon (IFN)-γ, IL-1β, IL-6, IL-10, and TNF-α, in mesenteric lymph nodes and the ileum [63]. Additionally, shorter villus height in the small intestine have been observed in pigs reared in LSC compared to those reared in HSC [64,81]. The change in intestinal integrity may explain the lower ATTD of dry matter, nitrogen, and gross energy in pigs housed in LSC [1,2]. Moreover, the physiological changes caused by LSC alter nutrient and energy metabolism, leading to shifts in AA and maintenance energy requirements. Kahindi et al. [80] reported that the standardized ileal digestible (SID) requirement of SAA increased by approximately 6–10% based on villus height and plasma urea nitrogen responses in weaned pigs. Similarly, Jayaraman et al. [81] estimated that SID Trp requirement is 4% higher when pigs are housed in LSC compared to HSC. Furthermore, van der Meer et al. [2] reported that pigs housed in LSC have 8% higher fasting heat production—the greatest portion of maintenance energy—than those in HSC.

As anticipated, LSC is able to modify the intestinal microbiome composition and functionality. A change in the abundance of lactic acid-producing bacteria has been noticeable in sanitary challenge studies. For example, Cho et al. [82] reported that LSC decreased the fecal abundance of the *Lactobacillaceae* family. Their findings were supported by Montagne et al. [61], who found lower *Lactobacillus* counts (colony forming unit) in feces of pigs reared in LSC. In another sanitary challenge study, Waititu et al. [63] reported lower *Bifidobacterium* spp. abundance in the cecum and colon, but no difference in *Lactobacillus*. Another study used metagenomic assay [62] and showed that the *Megasphaera* genus, known to be involved in lactate fermentation, was lower in the colon digesta of pigs reared in LSC. A reduced number of *Megasphaera* may be associated with lower concentration of lactic acid in the lumen. A general agreement exists that LSC results in greater protein fermentation. Cho et al. [82] reported that LSC increases the production of branched chain fatty acids in feces. Because branched chain fatty acids are produced from branched chain AA, an increase in the production of these fatty acids possibly reflects an increase in protein fermentation. Recent studies have consistently reported that LSC reduce butyrate concentrations in the colon [62], and feces [82]. Their findings were consistent with microbial assays in which the abundance of butyrate-producing bacteria, such as *Clostridiales* family XIII Incertae Sedis [82] and *Clostridium* IV and XIVa [63], decreased in LSC-challenged pigs. In contrast to butyrate, sanitary degradation generally increases total short chain fatty acids (SCFA) and volatile fatty acids (VFA) concentration. te Pas et al. [62] reported that LSC increases the abundance of *Lachnospiraceae* family in the colon digesta, which are known to be one of the major microbes involved in SCFA synthesis from complex polysaccharides. Montagne et al. [61] also reported an increase in VFA concentrations in the feces of nursery pigs housed in LSC. The authors postulated this phenomenon as the “hygiene hypothesis”, meaning that a clean environment can impede the optimal development of the immune system and microbiome establishment in young animals. However, LSC might decrease foregut digestion and increase undigested nutrients, thereby modifying hindgut fermentation patterns. Therefore, further studies are necessary to elucidate changes in fermentation patterns and microbial metabolites in relation to sanitary conditions.

Although the sanitary challenge model can be applied to nutritional studies, the model has been criticized due to the lack of reproducibility. While other immune challenge models can quantify the stimulation intensity (e.g., dose), quantifying sanitation level is difficult because the sanitary challenge model generates various stressors in different facilities and environments. Swine barns have different predominant pathogens and pathogenesis, causing the intensity of the stressors to vary depending on location. Furthermore, farms have different floor systems (e.g., slat, pit) which may affect the stressors’ intensity as this directly influences the quantity of manure accumulation and noxious gas exposure. These factors lead many studies to report no differences in growth performance or ISS between sanitary conditions. Therefore, to improve the reproducibility of the sanitation challenge model, details of experimental procedures and environments, including air quality, ventilation operation, ambient temperature, humidity, manure source and management, pen cleaning frequency, pen size, and floor design, should be provided.

### 3.4. Bacterial Lipopolysaccharide

Lipopolysaccharide is a component of the outer membrane of Gram-negative bacteria implicated in the pathogenesis of many disease states, causing endothelial cell injury and dysfunction. The LPS possesses a highly proinflammatory characteristic, which triggers the upregulation of cytokines, adhesion molecules, and tissue factor [83]. The model has been largely used in pigs to mimic systemic inflammation [68] and causes significant reduction in body weight gain [67,84]. It should be noted that the period after which LPS is administered may influence the response in growth performance of pigs, since tolerance may occur following multiple injections. A recent meta-analysis conducted by Rodrigues et al. [9] revealed a greater reduction in average daily gain in studies using LPS compared to studies using other challenge models (e.g., respiratory pathogens, low sanitary condition, environmental stress). This is consistent with sepsis and septic shock entailed by LPS administration mainly due to the overt cytokine production [85,86]. Among them, IL-6 circulating concentration, which is associated with fever, has been reported to increase after LPS injection [70,87]. Pastorelli et al. [8] reported in a meta-analysis that the reduction in performance during a LPS challenge was due to a depression in feed intake and not to increased maintenance requirements. More recently, these findings were contradicted by another meta-analytical approach which statistically contrasted different challenge models and showed that maintenance requirements had a substantial contribution to the decreased performance in LPS-challenged pigs [9]. Indeed, altered AA requirements have been demonstrated in multiple studies in LPS-challenged pigs, including Met [69], Thr [18], and Trp [65], which indicates that nutrient maintenance requirements/utilization efficiency play an important role in the observed reduction in performance.

Impairment in growth performance has been reported in weaned pigs, along with fever and increased production of major acute-phase proteins and cytokines (e.g., haptoglobin, tumor necrosis factor-α and interleukine-1 beta [71]. This is consistent with the findings of Xu et al. [72], who reported an increased plasma concentration of cortisol, prostaglandin E2 (PEG2), IL-6, TNF-α and IL-1β dramatically after LPS administration, which corroborates the systemic commitment after challenge. A recent study revealed the clear reduction in nutrient utilization efficiency in LPS-administered pigs, where not only growth performance but also the concentration of most microelements in feces and the expression of most microelement transport genes in the mucosa of the gastrointestinal tract were decreased after injection [73]. It is important to consider that LPS is a challenge model mainly used to mimic systemic activation of the immune system. However, since many pathogens and antigens are able to entail systemic response, it may be inferred that nutrient requirements are increased when there is a load to the production of immune cells and co-factors.

## 4. Nutritional Strategies for Improved Health Status

### 4.1. Low Protein, Amino Acid-Supplemented Diets

It is well known that high dietary protein (HP) content may have detrimental effects on gut health in pigs [88,89], which is presumably attributed to the indigestible fraction, available for microbial fermentation (i.e., protein not absorbed in the small intestine) and the potential for a high dietary protein level to support the proliferation of pathogenic bacteria, such as ETEC, by increasing the pH of the gut through the high buffering capacity of protein [90]. Studies have reported pro-inflammatory effects entailed by protein fermentation metabolites (e.g., branched-chain fatty acids, ammonia, biogenic amines, hydrogen sulfide, and phenolic and indolic compounds) including compromised colonic epithelial cell structure and metabolic functions, thinning of the mucus barrier, and increased colonic permeability [91,92,93] In this sense, studies have established a relationship between feeding HP diets and the incidence of post-weaning diarrhea in pigs [46]. Furthermore, it has been amply demonstrated that LP diets may improve gut health outcomes by suppressing the proliferation of pathogenic bacteria while promoting those with beneficial effects [94,95,96]. Therefore, the recommendation is that nutritionists should feed LP diets that are supplemented with crystalline AA to meet requirements for essential AA. It is thought that these diets reduce the amount of substrate (i.e., undigested protein) for pathogenic bacteria and production of harmful metabolites, leading to improved gut health and function in the postweaning phase [91].

In a recent review, Zhang et al. [89] reported that feeding HP diets evoked a shift in gut microbial composition favoring nitrogen-utilizing communities, including pathogenic groups, which is highly associated with incidence of diarrhea. Moreover, protein fermentability increased the concentration of (primarily) biogenic amines in the gut leading to impaired intestinal morphology, increased gut permeability, and increased pro-inflammatory cytokine concentration. While some studies have reported attenuation of diarrhea in post-weaned piglets fed LP diets [96,97], there is no consensus across studies. For example, Rodrigues et al. [10] reported minimal effects of dietary protein content when ST inoculated pigs were fed 16% or 20% protein diets. Conversely, Pollock et al. [98] reported that a HP diet aggravated the disturbances in the gastrointestinal environment entailed by ETEC challenge. This indicates that there are factors other than simply total dietary protein content (e.g., indigestible content, protein type) involved in the aggravation of post-weaning diarrhea. The contribution of protein fermentation metabolites to the negative effects on gut health are not fully elucidated [99] and there is still a lack of consensus on methodologies to evaluate and classify intestinal health [100].

It is important to highlight that the reduction in dietary protein must be accompanied by the supplementation of crystalline essential AA (EAA). For example, Yu et al. [101] fed weaned pigs diets with 20, 17, and 14% protein supplemented with Lys, Met, Thr, and Trp only. After a 45 d feeding period, the authors reported small intestine atrophy (e.g., decreased villus heights and lower ratios of villus height to crypt depth) and impaired pepsin activity in the stomach, suggesting an AA deficiency for intestinal development and enzyme activity. Likewise, Chen et al. [102] fed pigs a normal protein (18%), LP (15%), or extremely LP (12%) diet for 30 days and reported that the moderate protein restriction only positively modulated bacterial communities, increased the expression of tight junction proteins and enhanced epithelial cell proliferation in ileum. Spring et al. [103] further demonstrated the importance of supplementing very LP diets with adequate amount of crystalline AA. Supplementation of branched-chain AA attenuated the impairment in growth performance, energy balance, and metabolic and gut microbiome profile due to protein restriction (e.g., [104]).

### 4.2. Dietary Fiber Level and Source

Dietary fiber is a general classification which includes a broad spectrum of oligosaccharides and starch resistant to proximal intestine hydrolysis, as well as non-starch polysaccharides such as pectin, cellulose, hemicellulose, β-glucans and fructans. Subsequently, fiber sources can be classified according to their solubility, viscosity, physical structure, and water-holding capacity, which will combinedly determine their physiological role. In pigs, soluble dietary fiber is fermented primarily in the colon producing gases and several physiologically active by-products [105]. Insoluble fiber, in turn, generally increases diet bulkiness due to its metabolic inert characteristic [105]. Moreover, the well-known limited feed intake capacity of piglets may limit the ability of young animals to digest high fiber diets properly, which can be detrimental to growth performance and feed efficiency. For example, fiber may act as an antinutritional factor by decreasing nutrient digestibility and increasing endogenous mucin secretion, further aggravating the amount of undigested protein reaching distal parts of the gut [93] and decreasing AA availability for growth [106]. This is the reason why fiber levels are kept generally low in starter and nursery diets, as opposed to grower and finisher diets, where feed intake and digestion are not limiting factors.

In a recent review, Williams et al. [107] explored the categorization of dietary fibers into soluble or insoluble and discussed the importance of moving beyond this simple stratification. Basically, it is pivotal to understand how the microbial populations inhabiting the gut respond to a variety of fiber sources and levels, and how recommendations must be addressed in terms of fiber fermentability, rather than only solubility. In this sense, the inclusion of fermentable fiber sources and/or lowering dietary protein content can be used as strategies to potentiate beneficial metabolites while suppressing negative metabolites [88,91,108]. This is partly explained by a prioritization of energetic over protein fermentation by gastrointestinal microbes and an increased amino acid and ammonia incorporation into the microbial biomass [92,93,109]. However, it should be reiterated here that feeding soluble and rapid fermentable fiber sources during the immediate post-weaning period, especially weaning age is decreased and the sanitary condition around weaning is suboptimal, may lead to negative results, again, due to very limited feed intake capacity [110]. After two weeks post-weaning, when it is expected an improved adaptation of piglets to solid feed, a gradual inclusion of soluble and fermentable fiber may be advantageous for enhancing fermentation of nutrients and improving absorption of short-chain fatty acids in the large intestine mucosa.

Specifically, and unlike with protein, the inclusion of fermentable fiber in swine diets can have positive effects on the gastrointestinal environment as reviewed by Jha and Berrocoso [111]. The mechanisms underlying the positive effects of fermentable fiber in pigs is related to the formation of end products of fermentation (acetate, propionate, butyrate) and include improvement in colonic barrier function and immune/metabolism-related gene expression [112], maintenance of microbial community homeostasis [113], and attenuated release of inflammatory intermediates [114]. Regarding gut microbial populations, it has been suggested that dietary fermentable fiber improves microbiota stability and improves its diversity with promotion of proliferation of potentially beneficial microorganisms, including *Lactobacilli* and *Enterococci* [88,115,116], and suppression of potentially harmful populations, including *Clostridia* spp. and *Escherichia coli* [109,117]. Furthermore, evidence exists to show that the physical form of a fiber type may also influence its ability to mitigate the pathogenesis of *Escherichia coli* in swine. For instance, Molist et al. [118] reported that supplementing a nursery pig diet with coarse (1088µ) wheat bran prevented ETEC colonization of the small intestine and reduced severity of diarrhea compared with supplementing a finely (455µ) ground wheat bran in ETEC-challenged pigs. It is important to note that the impact of dietary fiber on host health and pathogen susceptibility have not been consistent across studies, possibly due to differences in fiber properties as well as fermentability potential of different sources [119,120]. Of note, feeding a high fiber diet may potentiate mucus secretion and epithelial cell sloughing which damages the gut architecture [121,122,123] and may increase animal susceptibility to pathogens. Indeed, Wellington et al. [49] reported decreased growth performance in *Salmonella*-challenged pigs fed high fiber diets, where fiber consisted of a mixture of soluble and insoluble sources. Moreover, in a subsequent study, Wellington et al. [124] showed improvements in barrier function of LPS-injected pigs when fed high fiber diets. Likewise, feeding non-starch polysaccharides to growing pigs increased gastrointestinal water secretion after infection with swine dysentery [125]. Finally, while the inclusion of beta-glucan in the diets improved growth performance of nursery pigs, there was an increased susceptibility to *Streptococcus suis* infection [126].

### 4.3. Diet Complexity

Diet complexity has long been discussed in swine nutrition because of its economic significance in nursery pig production. Diet complexity generally refers to dietary composition, where higher diet complexity indicates a greater number of ingredients in the diet and, generally, the inclusion of animal-based ingredients (e.g., fish meal, blood meal, plasma meal, and dairy products) as well as feed additives and antibiotics for the purposes of creating a diet with greater nutrient availability, reduction in anti-nutritional factors, and reduction in pathogen load. Animal-based ingredients, such as fish meal, whey, and blood meals are good protein sources with high-quality AA profiles that lack antinutritional factors (e.g., antigenic compounds and non-starch polysaccharides) [127,128]. Furthermore, the functionality of fish and blood meals (e.g., particularly spray-dried porcine plasma) has been reported in nursery pigs as enhancers of health status and growth performance [129,130]. The inclusion of dairy products, such as lactose, whey powder, whey permeates, and skim milk powder, provide a source of lactose, enhance palatability, and are thought to ease the transition to non-milk-based diets post-weaning [131].

Animal-based ingredients increase both the complexity and feed cost of the nursery pig diet. In addition, there has been recent social pressure to reduce the use of animal-based ingredients in livestock feed, with plant-based diets being considered by the public to be healthier and more welfare-friendly. Thus, efforts have been made to simplify diets during the nursery stage without compromising growth performance and productivity. In general, pigs fed a conventional, complex diet have greater body weight gain and feed intake in the post-weaning period than those fed a simple diet [131,132,133]. Furthermore, higher nutrient digestibility and daily energy intake have been observed in pigs fed complex diets compared to pigs fed a simple diet [134]. Interestingly, Wang et al. [135] reported a more balanced protein metabolism, as determined by urea nitrogen concentration, when pigs were fed a complex compared to a simple diet. However, these benefits do not last throughout the subsequent production period. For example, nursery diet complexity (simple vs. complex) does not alter carcass characteristics, including longissimus muscle quality, loin meat quality, and ham composition [132,136,137]. Therefore, lagged growth in nursery pigs resulting from a simplified, low-quality nursery diet can be compensated for during the re-alimentation period with improvements in feed efficiency, a phenomenon commonly referred to as compensatory growth.

Attempts have been made to mitigate the negative effects of a simple diet on the immune system and intestinal microbiota. Koo et al. [134] showed that dietary feed enzyme supplementation to a simple diet can be beneficial in terms of nutrient and energy digestibility and intestinal morphology. In another study by Koo et al. [50], supplementation of 0.1% of L-Thr to the simple diet increased the number of jejunal goblet cells wherein mucins are produced. The authors postulated that this benefit led to a reduction in ammonia nitrogen concentrations in the jejunum and downregulated intestinal inflammation, thereby increasing the villus height to crypt depth ratio. However, the supplementation of L-Thr to the simple diet did not restore the increased IL-6 in the serum—a biomarker of systemic inflammation—to the levels typical of a complex diet. Therefore, to successfully replace a complex diet with a simple diet in commercial nursery production, nutritional strategies that can prevent systemic metabolic changes should be studied.

### 4.4. Functional Amino Acids

As previously stated, when the immune system is stimulated after an injury or infection there is a prioritization of AA utilization for the immune response at the expense of growth [138]. Moreover, the reduced feed intake, which commonly occurs as a result of ISS, exacerbates the reduction in supply of AA, further impairing lean tissue deposition [28,139,140]. In this sense, it is reasonable to infer that a significant amount of muscle protein, as the largest body AA pool, will be mobilized in order to meet changing requirements associated with the immune response [138]. However, as shown by Reeds et al. [138], there is a notable difference between the AA profile of muscle protein and the profile of many important acute-phase proteins involved in the immune response. This indicates an imbalance which, unless supplemented through the diet, will trigger significant whole-body AA catabolism and reduction in body protein deposition [138,141]. For example, pigs fed a SAA-free diet had lower intestinal Cys concentration, associated with reduced jejunal goblet cell number, which may indicate insufficient secretion of Cys-rich mucins involved in the intestinal innate immune response [142,143]. Albumin is a Cys-rich acute-phase protein, with production of 1 g of albumin requiring approximately 6 g of muscle protein breakdown in the absence of another source of Cys. Likewise, neonates, highly dependent on the innate defenses of mucus [144], show impaired mucin production and gut function when fed a Thr-deficient diet [145]. Traditionally, nutritionists have defined AA essentiality based on growth performance outcomes and there is a lack of information in the literature regarding the role they perform on the modulation of immune response, anti-oxidative defense, and recovery from injuries [146]. However, there is growing interest in the ‘functional’ roles AA have beyond their role as constituents of lean gain [32].

Liu et al. [147] conducted a dose–response study with 21 d old piglets investigating the ratio between SAA and Lys, testing the content of 70, 85, 100, 115, or 130% of the SAA: Lys ratio recommended for growth. The authors reported a downregulation of genes related to inflammation (e.g., TNF-a, transforming growth factor (TGF)-β, and IL-1β) as SAA intake increased from 0.63%, suggesting a functional role of SAA even considering weanling, presumably healthy piglets. Similarly, Yan et al. [148] fed weanling pigs a low (0.53%) or high (0.85%) SAA diet for one week and clearly showed that the increased intake enhanced jejunal cell proliferation and function, mainly through improved antioxidant capacity, and Wnt/β-catenin and mTOR signaling pathway. Weanling pigs fed increasing levels of SID Trp: Lys ratios (16.1%, 18.6%, 20.3%, 22.9%, and 24.6%) and challenged with *Escherichia coli* K88 had optimal performance when fed 21% and showed increased expression of the anti-inflammatory IL-10 with increasing ratios [81].

Recent studies have shown that endogenous (kynurenine, serotonin, and melatonin) and bacterial (indole, indolic acid, skatole, and tryptamine) Trp metabolites play an important role in gut microbial composition and metabolism, immune response of the host, and host-microbiome interaction [149]. Moreover, it has been highlighted that stress and disease, including irritable bowel syndrome and inflammatory bowel disease, directly alter Trp metabolism and disturb the Trp–microbiome–immune system interaction in the gut. It has been shown that higher Trp intake resulted in a number of beneficial effects in the piglet gastrointestinal tract, namely: (1) enhanced microbiome diversity, (2) decreased abundance of opportunistic bacteria, and (3) increased mucosal IL-8 mRNA level, and zonula occluden (ZO)-1 [150]. Koo et al. [50] reported an increased villus height and goblet cell density, and a higher expression of jejunal occluden and downregulation of IL-6 in pigs fed diets with 115% compared to 100% of the requirement for SID Thr. This is in line with recent findings showing that supplemental Thr (120% of requirements) improved growth performance in ST pigs fed low fiber (LF) diets [49], and increased fecal mucin output in ST pigs fed high fiber (HF) diets [124]. There is also recent evidence showing that the combination of AA can perform functional properties in pigs subjected to immune challenge. Rodrigues et al. [10] fed a basal or a FAA (Thr, Met, and Trp at 120% of requirements) profile to 14 kg pigs for 14 d, divided in pre- and post-inoculation with either saline or ST. Pigs inoculated with ST and fed the FAA profile had greater weight gain, improved antioxidant defenses, attenuated acute-phase response, and reduced pathogen shedding and colonization. In a subsequent study, the authors reported that the previously positive results were enhanced when pigs were fed the FAA profile for a longer period (e.g., 2 weeks) before ST inoculation, with no effects of diets during the pre-inoculation phase [11]. These results suggest that immune status, gut health, and overall pig robustness may be improved with the supplementation of FAA [151,152,153], which becomes particularly important in diseased pigs. Interestingly, a recent study revealed that FAA supplementation triggered a positive response in mitigating the effect of enteric disease challenge in normal birth weight (NBW), but not low birth weight (LBW) pigs [154]. Additionally, FAA supplementation partially attenuated the detrimental effects of plant-based (PB) diets on the response of pigs to ST challenge, while FAA supplementation had minimal effects in ST-challenged pigs fed animal-based (AB) diets [155].

### 4.5. Organic Acids

Among various feed additives, OA have been vigorously researched in swine nutrition in relation to gastrointestinal health. Organic acids can be broadly classified into three categories based on the carbon chain: SCFA (e.g., formic acid, acetic acid, propionic acid, butyric acid), medium chain fatty acids (MCFA; e.g., caproic acid, caprylic acid, capric acid, lauric acid), and tricarboxylic acids (e.g., citric acid, fumaric acid, and malic acid). Most importantly, OA possess bacteriostatic and bactericidal actions. Undissociated forms of OA can diffuse across the bacterial cell membranes, where they are dissociated inside the cells and release H+ ions, thereby disrupting the acid-base balance and vital metabolic pathways of microbes [156]. However, the antimicrobial efficacy of OA against pathogens varies depending on their physicochemical properties (e.g., pKa, lipophilicity, and solubility) and the target microbes (e.g., the structure of cell walls or membranes) [157]. For example, SCFA have shown strong efficacy against Gram (−) bacteria, including *E. coli* and *Salmonella* spp., while MCFA have shown strong efficacy against Gram (+) bacteria, such as *C. perfringens* and *Steptococcus* spp. [157,158]. Therefore, combining different OA may be critical for maximizing efficacy against different pathogens. The present review focuses on the effects of dietary OA supplementation in immune-challenged pigs. This discussion will help introduce OA practicality on farms, in which the environment is more stressful to pigs than the experimental environment.

Unlike the straightforward efficacy found in vitro, in vivo studies have shown controversial results in ST or ETEC-challenged pigs. Gebru et al. [159] reported that dietary microencapsulated OA (citric acid, fumaric acid, malic acid, and phosphoric acid in a 2:2:1:1 combination) supplementation at 0.2% decreased the fecal shedding of ST and improved growth performance for the 4-week experimental period in growing pigs (initial BW: 38.7 kg). Similarly, Calveyra et al. [160] found that OA supplementation decreased the number of *Salmonella* spp. in the feces of growing pigs. In contrast, other studies in nursery pigs failed to find the benefits of OA supplementation alone (0.8%, 1%, or 2.58 mL/L of water) on the microbial profile or ST shedding in ST-challenged nursery pigs [161,162,163]. Interestingly, Fabà et al. [163] reported that a combination of OA with mannan-rich hydrolyzed copra meal or fermented rye reduced the shedding of ST in ST-challenged pigs. This may suggest that OA alone are not sufficient to control the pathogenicity of ST in nursery pigs. On the other hand, the efficacy of OA against ETEC in nursery pigs is much more apparent. Dietary OA supplementation reduced the incidence of diarrhea or fecal scores in ETEC-challenged pigs [164,165]. The immunomodulatory effects of OA have also been observed in ETEC-challenged pigs. Organic acids supplementation decreased concentration of pro-inflammatory cytokines, such as IL-1β, IL-6, TNF-α, and IFN-γ, in plasma to levels comparable to antibiotics supplementation [166]. Similarly, Jiménez et al. [167] reported that OA supplementation decreased the number of inflammatory cells in the jejunal and ileal lamina propria, which had been elevated by ETEC inoculation.

Further benefits of OA on intestinal and hepatic redox status were reported based on an increase in GSH and ferric-reducing ability potential and a decrease in thiobarbituric acid reactive substances (TBARS) concentrations in the intestine or liver of pigs fed an OA-supplemented diet [167]. However, the effect of OA on intestinal microbial composition seems to be inconsistent. For example, Han et al. [168] performed 16s rRNA sequencing with ileum digesta samples and showed that, based on the alpha diversity indices (Chao1, ACE, and Shannon indices) and shift in *Firmicutes* and *Proteobacteria* abundance, pigs fed an OA-supplemented diet had a more diverse and stabilized microbiome composition than control diet-fed pigs. In contrast, Ren et al. [166] failed to find the benefits of OA mixture supplementation on fecal counts of total coliforms and *Lactobacillus*. Stensland et al. [169] reported no effects of OA supplementation on the counts of total *E. coli*, F4 *E. coli*, *Enterobacteriaceae*, and *Lactobacillus* spp., but they did find an increase in total VFA concentration in the feces of pigs fed an OA-supplemented diet. Differences in ETEC inoculation dose, sampling time, and analysis methods may have contributed to the contradictory results among these studies.

### 4.6. Plant Secondary Metabolites

Due to the ban on antimicrobial growth promoters, growing interest in organic livestock farming has accelerated the use of plant secondary metabolites (PSM), often called phytogenic feed additives. Plants have evolved to adapt to the environment, which for some plants involves the production of special metabolites that serve as a defense mechanism against exogenous stressors such as germs and oxidative damage [170]. Various PSM can be beneficially ingested by animals, providing immunomodulatory, antioxidative, anti-inflammatory, antifungal, antiviral, antibacterial, and anti-diarrheal effects [171]. Plant secondary metabolites are broadly classified into terpenes (e.g., carvacrol, thymol), phenolics (e.g., eugenol, resveratrol, quercetin, tannins), N-containing compounds, and S-containing compounds (e.g., alliin and allicin). Phenolics are further divided into polyphenols (e.g., tannins and flavonoids), phenolic acids (e.g., benzoic acid, ferulic acid, gallic acid, vanillin), and miscellaneous (e.g., lignans, resveratrol) [170].

In pig nutrition, various sources of PSM have been studied. Generally, PSM have been delivered through plant extracts and agro-industrial by-products (e.g., grape pomace). These products usually contain a mixture of PSM and have reduced risk of causing the emergence of resistant bacteria due to their compositional and chemical complexity [172]. However, because their bioactive components and profiles change depending on the plants’ maturity, harvesting time, and weather, producing qualitatively consistent products is difficult [173], which creates challenges in diet formulation. Advanced technologies have allowed some PSM to be chemically synthesized and these products are commonly called nature-identical compounds [174]. These synthesized compounds (e.g., thymol and vanillin) can be precisely supplemented to swine diets and synergically combined with other feed additives.

Essential oils, which are either terpenes or phenolics, are concentrated, highly volatile hydrophobic liquids. They are usually extracted from plants, but several naturally identical EO are commercially available. Because of their phenolic ring, or capacity to disturb microbial membranes and intracellular homeostasis, EO usually show antimicrobial, anti-inflammatory, and antioxidative properties [175]. Dietary EO blends, such as thymol and cinnamaldehyde, improve antioxidant capacity by enhancing antioxidant enzymes, including superoxide dismutase and catalase in the serum or mucosa of weaned piglets [176,177]. Improved digestibility of dry matter, gross energy, crude protein, and AA has been consistently reported in pigs fed EO blend-supplemented diets [178,179,180]. In a study by Ruzauskas et al. [181], pigs received 3 tablets daily, each of which contained 986 mg of oregano extract, 3 mg of peppermint EO, and 7 mg thyme. These pigs had a higher abundance of bacteria considered probiotic, such as *Lactobacillus*, *Bacillus*, and *Bifidobacterium*, in the gut (i.e., ileum, cecum, and colon) compared to control pigs that did not receive EO. Similarly, a reduction in *E. coli* or *Enterobacteria* counts and increase in *Lactobacillus* counts in feces [178] and the jejunum and cecum digesta [182] was found in pigs fed EO-containing diets compared to pigs fed a control diet. The microbial modulation by dietary EO possibly leads to a reduction in the incidence of post-weaning diarrhea [178,180,182]. However, the anti-diarrheal effect seems to be dependent on the dose. Cairo et al. [182] reported that 0.15% of red pepper EO reduced diarrhea incidence by 43% compared to a control group, but 0.1% supplementation increased the incidence by 21%.

Dietary EO supplementation to a swine diet has been often tested in combination with other compounds, particularly OA, as EO and OA have better efficacy against Gram (+) and Gram (−) bacteria, respectively [44,157,183]. Apart from EO, plant extracts as a source of polyphenolic compounds and polyphenol-rich ingredients have been investigated frequently. Coddens et al. [184] reported that cranberry extracts rich in proanthocyanin could inhibit the adhesion of F4+ and F18+ *E. coli* on the ileum in vitro. They found that supplementing the diet (0.1%) and water (1 g/L) with cranberry extracts reduced the diarrhea score and F18+ *E. coli* shedding in nursery pigs. Similarly, Xu et al. [72] found that 250 mg/kg of holly polyphenols elevated jejunal and disaccharides (sucrase and lactase) activity, upregulated tight junction proteins in the ileum (claudin-1 and occludin), and decreased pro-inflammatory cytokine contents in the plasma. Furthermore, holly polyphenol supplementation increased *Lactobacillus* in the cecum and colon, regardless of LPS challenge, and it restored the cecal abundance of *Prevotella*, a major dietary fiber fermenter, which is suppressed by LPS administration.

Grape by-products can also be beneficially fed to pigs as a polyphenol source [185]. For example, Kafantaris et al. [186] reported that the dietary inclusion of fermented grape pomace at 48.5% enhanced the antioxidant defense system based on the levels of GSH, total antioxidant capacity, TBARS, and protein carbonyls in various organs, including the liver and pancreas of nursery pigs. Additionally, the inclusion of grape pomace beneficially modified the fecal bacteria composition by increasing *Bifidobacterium* and lactic acid bacteria counts and reducing *Enterobacteriaceae* counts. Gessner et al. [187] found that supplementation of grape seed and grape marc meal extracts in a nursery diet at 1% suppressed the activity of NF-κB, an inducible transcription factor for inflammatory responses and the expression of inflammatory cytokines in the duodenal mucosa of nursery pigs.

Nevertheless, the efficacy of PSM follows the hormesis concept, meaning that an excessive amount of PSM may serve as an antinutritional factor, stimulating the immune system and reducing nutrient digestibility. Therefore, dietary supplementation levels should be optimized for the promising PSM before it is commercialized.

## 5. Conclusions

Swine production systems expose pigs to a variety of immune-stimulating agents, which impact nutrient utilization in the pig to support of the immune response. With reductions in antibiotic use, there is an increased need and focus on research into potential nutritional strategies to maintain both animal health and performance when pigs are exposed to immune stimulating conditions. There is robust evidence showing that feeding LP, AA-supplemented diets may overcome the negative effects of undigested protein on gastrointestinal health. Moreover, research has increasingly highlighted AA not only as building blocks for muscle protein, but also as functional agents. Supplementation with key FAA has been shown to enhance pig performance and immune status. Feeding different fiber sources and levels may improve gastrointestinal health by ameliorating intestinal development and renewal, and positively modulating the microbiota. Finally, and particularly for the immediate post-weaning period, increasing diet complexity by incorporating highly digestible (i.e., animal-based) ingredients, and supplementing diets with organic acids and/or plant secondary metabolites may improve the pig’s ability to cope with the weaning transition. Overall, nutritionists will need to incorporate animal health requirements into future nutrition programs and consider multiple nutritional strategies depending on the specific conditions to which pigs are exposed in order to optimize both immune status and productivity of pigs.

## Figures and Tables

**Table 1:** Mechanisms through which challenge models impact nutrient digestion and metabolism and growth performance.

Item.	Age (Weight)	Agent	Effect	Reference
Enteric pathogen challenge	28 d (8.5 kg)	ETEC	Increased gut permeability, decreased small intestinal villus height	[44]
	21 d (6.9 kg)	ETEC	Increased fecal ETEC score, decreased amino acid digestibility, increased pH in the caecum and proximal colon	[46]
	21 d (6.4 kg)	ETEC	Shorter duodenal villus height and deeper jejunal crypt depth	[50]
	-	ETEC	Production of toxins (heat-labile and heat-stable) in the small intestinal epithelial cell layer	[33]
	(4.9 kg)	ETEC	Intestinal damage and reduced nutrient digestibility	[43]
	21 d (7.3 kg)	ETEC	Impaired mucosal immune function, intestinal morphology and integrity	[47]
	-	ST	Injection of *Salmonella* invasion protein into the intestinal epithelial cells, leading to cytoskeletal rearrangement	[38]
	(13.9 kg)	ST	Activation of acute-phase response, imbalance to antioxidant defense systems	[10]
	(22.6 kg)	ST	Decreased serum albumin concentration and increased haptoglobin concentration	[49]
Respiratory pathogen challenge	-	AP MYC	Chronic pleurits	[51]
	-	AP	Cranio-ventral pulmonary consolidation lesions	[52]
	-	AP MYC	Pleuritis and pneumonia	[53]
	-	MYC	Exfoliation of epithelial cells, increased peribronchiolar and perivascular monocellular cell accumulation	[54]
	75 d	MYC	Lung consolidation	[55]
	-	PCV2	Decrease in lymphocytes followed by an increase in neutrophils	[56]
	(13.1 kg)	PRRSV	Decreased expression of markers of skeletal muscle synthesis and increased liver glycogenolysis	[57]
	(33.6 kg)	PRRSV	Reduced digestibility of dry matter, energy and nitrogen, whole body accretion of lean, protein and fat	[12]
Degradation of sanitary condition	21 d (6.2 kg)	LSC	Lower apparent ileal and total tract digestibility of dry matter and lower apparent total tract digestibility of protein	[58]
	21 d	LSC	Decreased villus height and crypt depth in ileum	[59]
	28 d (7.8 kg)	LSC	Greater plasma haptoglobin concentration and decreased Trp concentration	[60]
	28 d	LSC	Increased diarrhea occurrence, higher counts of *Lactobacillus* and *Enterobacteria* and lower anaerobic sulfite bacteria, increased fecal volatile fatty acid concentration	[61]
	7 d	LSC	Higher abundance of lactate fermenting microbes, altered amino acid metabolism, immune response, and microbiome-specific metabolites in blood	[62]
	24 d	LSC	Increased serum haptoglobin, IgG antibody titers against keyhole limpet hemocyanin, pleuritis scores at slaughter	[1]
	70 d	LSC	Reduced incremental protein efficiency	[2]
	21 d	LSC	Impaired ileal immune response	[63]
	18 d (6.0 kg)	LSC	Shorter villous length and lower crypt depth	[64]
Bacterial lipopolysaccharide	(20.0 kg)	LPS	Increased plasma concentrations of acute-phase proteins and white blood cell counts, and decreased plasma albumin	[65]
	(21.3 kg)	LPS	Increased serum amyloid concentration	[66]
	14 d (4.5 kg)	LPS	Increased crypt depth of the duodenum and decreased ratio of villus height to crypt depth of the ileum	[67]
	-	LPS	Anorexia and fever	[68]
	(20.0 kg)	LPS	Increased eye temperature, and disturbed N balance	[69]
	-	LPS	Increase in interleukin-6 and tumor necrosis factor activity in plasma	[70]
	(20.5 kg)	LPS	Altered serum concentration of albumin, haptoglobin, fibrinogen, whole blood white blood cell, and platelet count	[18]
	24 d (6.6 kg)	LPS	Increased rectal temperature, and serum concentrations of haptoglobin, tumor necrosis factor-α and interleukine-1 beta	[71]
	-	LPS	Increased plasma concentrations of cortisol, prostaglandin E2, interleukin-6, tumour necrosis factor-α and interleukin-1β	[72]
	25 d	LPS	Decreased apparent digestibility of crude fat and microelement absorption	[73]

ETEC = enterotoxigenic *Escherichia coli*, LSC = low sanitary condition, MYC = *Mycoplasma hyopneumoniae*, PCV2 = Porcine circovirus type 2, PRRSV = Porcine reproductive and respiratory syndrome virus, ST = *Salmonella* Typhimurium.

## Data Availability

Not applicable.
